# Differential Effects of High-Carbohydrate and High-Fat Diet Composition on Metabolic Control and Insulin Resistance in Normal Rats

**DOI:** 10.3390/ijerph9051663

**Published:** 2012-05-04

**Authors:** Jorge L. Ble-Castillo, María A. Aparicio-Trapala, Isela E. Juárez-Rojop, Jorge E. Torres-Lopez, Jose D. Mendez, Hidemi Aguilar-Mariscal, Viridiana Olvera-Hernández, Leydi C. Palma-Cordova, Juan C. Diaz-Zagoya

**Affiliations:** 1 Centro de Investigación, DACS, Universidad Juarez Autónoma de Tabasco (UJAT), Villahermosa, Tabasco 86150, Mexico; Email: iselajua22@yahoo.com.mx (I.E.J.-R.); jorge.torres@ujat.mx (J.E.T.-L.); maestra20042002@yahoo.com.mx (H.A.-M.); viryolvera11@gmail.com (V.O.-H.); lady_simpson_8@hotmail.com (L.C.P.-C.); zagoya@unam.mx (J.C.D.-Z.); 2 División Académica de Ciencias Agropecuarias, UJAT, Villahermosa, Tabasco 86280, Mexico; Email: sabina52@hotmail.com; 3 Unidad de Investigación Médica en Enfermedades Metabólicas, Hospital de Especialidades, CMN, Siglo XXI, IMSS, Mexico D.F. 06703, Mexico; Email: mendezf@unam.mx

**Keywords:** metabolic control, high-carbohydrate diet, high-fat diet, resistant starch, insulin resistance

## Abstract

The macronutrient component of diets is critical for metabolic control and insulin action. The aim of this study was to compare the effects of high fat diets (HFDs) *vs.* high carbohydrate diets (HCDs) on metabolic control and insulin resistance in Wistar rats. Thirty animals divided into five groups (n = 6) were fed: (1) Control diet (CD); (2) High-saturated fat diet (HSFD); (3) High-unsaturated fat diet (HUFD); (4) High-digestible starch diet, (HDSD); and (5) High-resistant starch diet (HRSD) during eight weeks. HFDs and HCDs reduced weight gain in comparison with CD, however no statistical significance was reached. Calorie intake was similar in both HFDs and CD, but rats receiving HCDs showed higher calorie consumption than other groups, (*p* < 0.01). HRSD showed the lowest levels of serum and hepatic lipids. The HUFD induced the lowest fasting glycemia levels and HOMA-IR values. The HDSD group exhibited the highest insulin resistance and hepatic cholesterol content. In conclusion, HUFD exhibited the most beneficial effects on glycemic control meanwhile HRSD induced the highest reduction on lipid content and did not modify insulin sensitivity. In both groups, HFDs and HCDs, the diet constituents were more important factors than caloric intake for metabolic disturbance and insulin resistance.

## 1. Introduction

The importance of nutrient composition has been more widely recognized on account of dysmetabolic diseases such as obesity and diabetes, which are directly related to the incidence of cardiovascular disease. Obesity is considered a serious disease affecting a large population worldwide [1,2]. Insulin resistance (IR) is considered the key mechanism unifying obesity, diabetes and heart disease [3]. In the course of months or years, IR is followed by the increase in β-cell insulin secretion and by several complications known as the insulin resistance syndrome, which is associated with dyslipidemia, hypertension, hyperglycemia and cardiovascular disease [4]. Thus, efforts are continuously underway to prevent obesity in the population. Although weight gain is ultimately the result of an overall positive energy balance, the environmental and genetic interplay that accounts for the dramatic rise in obesity is not fully understood. Although the traditional weight loss approach advises a high carbohydrate low fat diet, a very low carbohydrate high fat diet has been suggested to have greater effectiveness in weight loss and metabolic improvement [5,6,7]. One potential problem associated with chronic ingestion of a low-carbohydrate diet is that it usually contains a high percentage of fat to compensate for carbohydrate-calorie reduction, and most in the form of saturated fat [8]. High-saturated fat diet in humans is known to be associated with high risk of diabetes and cardiovascular disease [9,10]. In rats a high-saturated fat diet is used as a diabetogenic factor increasing insulin and lipid levels and it has been shown to induce severe insulin resistance in skeletal muscles [11,12,13]. In general, most studies on nutrition research have been focused on studying food fat content and less have been concentrated on the importance of carbohydrate composition for glucose homeostasis and insulin resistance [14]. The disadvantage of consuming simple sugars (like monosaccharides and disaccharides) was understood and it was recommended to include complex carbohydrates in the diet. This point of view changed in recent years, because the digestibility of complex carbohydrates and dietary fiber, became known, the concept of resistant starch emerged, and the concept of glycemic index also emerged [15,16,17].

In 2009, the Codex Alimentarius Commision’s Committee on Nutrition and Foods for Special Dietary Uses, adopted a new definition of dietary fiber as “carbohydrate polymers with 10 or more monomeric units, which are not hydrolized by the endogenous enzymes in the small intestine of humans” [18]. Thus, resistant starch (RS), which is the portion of starch that resists digestion in the small intestine, is a recently recognized source of fiber and it is classified as a fiber component which reaches partial or complete fermentation in the colon.

Unripe-banana is known to be the non-manufactured food with the highest resistant starch content. The native banana starch (NBS) obtained from unripe banana (*Musa cavendish* AAA) in Tabasco, Mexico exhibits a low glycemic index (22.4) and a high resistant starch content (32%). In previous studies this product has demonstrated beneficial effects on body weight reduction and glucose homeostasis in animal models and humans [19,20,21].

Although the differential effects of high-saturated *vs.* high-unsaturated fat diets have become known, fewer studies have been focused on the differential effects of complex carbohydrates on metabolic control. In addition, there are no studies comparing unbalanced diets composed of normal banana starch or digestible starch with those containing high fat on metabolic control and insulin resistance. The aim of this study was to compare the effects of high-carbohydrate diets containing NBS or digestible starch with those containing high fat on the metabolic control and insulin resistance in normal Wistar rats.

## 2. Material and Methods

### 2.1. Materials

Casein was purchased from Novag Infancia (Mexico City), Lard (Keken) was obtained from Grupo Porcicola Mexicano (Yucatan, Mexico). Extra virgin olive oil was obtained from Carbonell (Spain) imported by Arroz SOS de Mexico, 13.3% saturated fatty acids, 6.6% poliunsaturated fatty acids (PUFAs) and 73.3% monounsaturated fatty acids (MUFAs). Corn oil (Mazola) from Alimentos Capullo. Corn starch was purchased from Unilever de Mexico. Native banana starch was provided by the Centro de Investigaciones Agropecuarias, Universidad Juarez Autonoma de Tabasco, situated in Teapa, Tabasco. NBS was obtained from unripe (green) bananas (*Musa cavendish* AAA) by a previously described procedure [21]. Resistant starch content measured according to the Association of Official Analytical Chemists (AOAC) 2002.02 method (Megazyme International Ireland Ltd was found to be 34% on a dry weight basis and the glycemic index was 22.4.

### 2.2. Animals and Diets

Male Wistar rats (n = 30) were obtained from the breeding colony maintained by the Unidad de Producción, Cuidado y Experimentacion Animal (UPCEA), from the División Académica de Ciencias de la Salud (DACS), Universidad Juárez Autonoma de Tabasco (UJAT), verified by the Secretaria de Agricultura, Ganaderia y Recursos Pecuarios (SAGARPA 2005). All procedures were subject to regulations of animal experimentation from the Norma Oficial Mexicana NOM-062-ZOO-1999, and the International Guide for Caring and Use of Laboratory Animals NRC 2002. Rats at the age of 7 weeks and 180–200 g body weight were maintained at their housing conditions with a controlled humidity (55%) and a 21 ± 1 °C temperature, 12–12 h light-dark. Animals were switched to one of the five different experimental diets: Control diet (CD, 2018S Teklad Global 18% Protein Rodent Diet from Harlan Laboratories), high-saturated fat diet (HSFD), high-unsaturated fat diet (HUFD), high-digestible starch diet (HDSD) and high-resistant starch diet (HRSD). Multiple cages were used being six animals by group. In this way, two different high-fat diets (HFDs) and two high-carbohydrates diets (HCDs) were administered in comparison with the control diet (CD). The 2018S Teklad Rodent Diet contained as percentage of calories 18.6% proteins, 44.2% carbohydrates and 6.2% fat. The energy content of this diet was 3.8 Kcal/g. The HFDs consisted of a high-saturated fat diet (HSFD) containing 58% lard and the high-unsaturated fat diet (HUFD) containing 58% olive oil. The HCDs consisted of a high-digestible carbohydrate diet (HDCD) containing 67% digestible corn starch and the high-resistant starch diet with 67% native banana starch (NBS). Animals were given free access to water and diets during the eight week duration of the experiments. Table 1 shows the details of diet composition. Diets were freshly prepared each day as pellets and the ingredients were separately stored at 4 °C. The energy intake was measured for each group from the 24 h food intakes calculated from the differences in daily food weighs. Individual body weight of rats in each group was assessed twice a week.

**Table 1:** Diet composition.

		HSFD	HUFD	HDSD	HRSD
		g/Kg	g/Kg	g/Kg	g/Kg
Protein					
	Casein	254.1	254.1	212.9	212.9
Lipid					
	Corn oil			11.1	11.1
	Olive oil		364.5		
	Lard	364.5			
Carbohydrates					
	Corn starch	305	305	675	
	Banana starch				675
	Sucrose	33.8	33.8	67	67
Vit. & Min.		15	15	15	15
Caloric value (Kcal/g)		5.6	5.6	3.9	3.9

CD, control diet; HSFD, high-saturated fat diet; HUFD, high-unsaturated fat diet; HDSD, high-digestible starch diet; HRSD, high-resistant starch diet. High-fat diets contained as percentage of calories 18% proteins, 24% carbohydrates, 58% fat and high-carbohydrate diets: 21.8% proteins, 75.6% carbohydrate, 2.5% fat.

### 2.3. Oral Glucose Tolerance Test

Two days before finishing experimentation (day 54) an oral glucose tolerance test (OGTT) using glucose 1 g/Kg BW was performed after a 12 h fasting period. Blood glucose concentration from the tail vein was measured using the Accu-Chek blood glucose meter (Roche Diagnostics, Mainz-Hechtsheim, Germany) at 0, 30, 60, 90 and 120 min.

### 2.4. Rat Euthanization and Sample Collection

At the end of the 8-week dietary period food was removed at the end of the dark period (0700) and animals left 12 h fasting until 0800 to begin experimentation. Rats were anesthetized with intraperitoneal pentobarbital sodium (60 mg/Kg body weight). Blood samples were obtained from the jugular vein and were rapidly centrifuged, plasma was either immediately used or stored at −20 °C during 7 days. The liver was totally removed, weighted and used for biochemical determinations.

### 2.5. Biochemical Measurements

Glucose, cholesterol, low-density lipoprotein cholesterol (LDL-C), high-density lipoprotein cholesterol (HDL-C) and triglycerides were analyzed by using an Architect Clinical Chemistry Autoanalyzer System from Abbott Clinical Chemistry (Chicago, IL, USA). Insulin was measured by the Rat Insulin ELISA kit (IBL International GMBH, Hamburg, Germany). Insulin resistance was estimated according to the Homeostasis Model Assessment (HOMA-IR) which was calculated by the product of the fasting concentrations of glucose (mg/dL) and insulin (μU/mL) divided by 405 [22]. Hepatic lipids were extracted using the Folch method [23]. Lipid extracts were resuspended for measurements of cholesterol and triglycerides by immunoenzymatic assays.

### 2.6. Statistical Analysis

Data are expressed as mean ± SEM. One-way analysis of variance (ANOVA) was used to compare data among groups. When a statistically significant difference was encountered, a Bonferroni’s multiple comparison test was performed. The total OGTT area under the curve (AUC) was calculated using the trapezoid method. Statistical significance was defined as *p* < 0.05. The calculation was performed using GraphPAD PRISMA software version 5.01 (Graphpad Software, Inc., San Diego, CA, USA).

## 3. Results

### 3.1. Body Weight and Energy Intake

**Figure 1:**
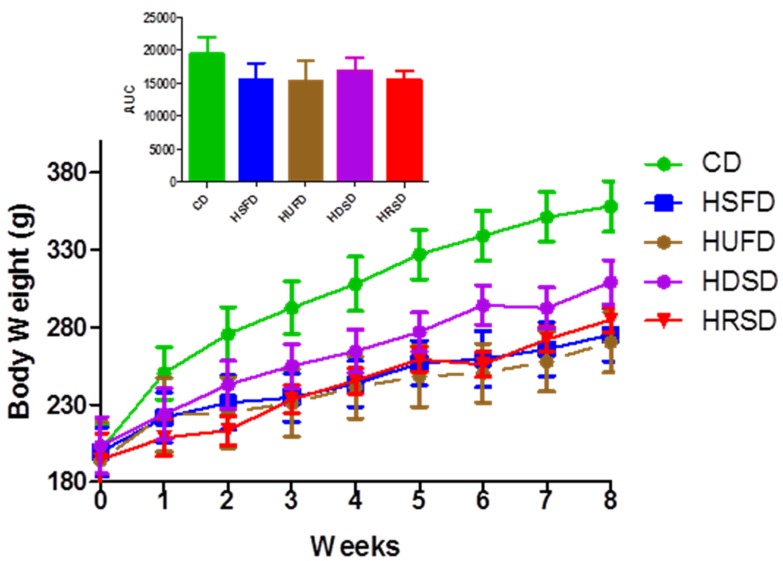
Temporary course of body weight gain during 8-week treatment in the experimental groups. Control diet (CD), high-saturated fat diet (HSFD), high-unsaturated fat diet (HUFD), high-digestible starch diet (HDSD), high-resistant starch diet (HRSD). No significant differences between body weight AUCs were observed between groups. ANOVA and Bonferroni’s multiple comparison test.

Figure 1 shows the body weight changes through the experimental period and their respective areas under the curves (AUCs). At the onset of the experiment, all the animals included in the five study groups had similar body weights. Body weight gain in the CD group was progressively higher than in the other groups, but no statistically significant differences were observed when the AUCs for body weight were compared between groups (Figure 1). At the end of the experimental period, the CD group showed body weight gain higher than the HSFD, HUFD and HRSD groups (*p* < 0.05), however, no significant difference was reached with the HDSD. No significant difference between HFDs and HCDs groups were observed. Table 2 presents the data of final body weight and the average of total energy intake over the experimental period by group of animals. Animals receiving HFDs showed total caloric intake similar to the CD group but lower than the HCDs (*p* < 0.001). Despite the higher caloric intake in the HCDs groups relative to the HFDs groups, no differences in body weight gain were observed among them. The HRSD group showed the highest caloric intake in comparison with the other groups, including the HDSD (*p* < 0.001). There were not significant differences in body weight gain and total caloric intake between the HSFD and the HUFD groups.

### 3.2. Glycemic Control

Table 3 shows the effects of experimental diets on parameters related to glycemic control. HUFD group exhibited the lowest fasting glycemia levels among all groups, however, it was only significant when compared to the HRSD group.

**Table 2:** Final weight gain and whole energy intake in rats fed a control, HFDs and HCDs during 8 weeks of experimental period.

	CD	HSFD	HUFD	HDSD	HRSD
**Body weight (g)**	307 ± 16.7 ^a,B,C^	247 ± 8.0 ^a^	242 ± 7.7 ^B^	268 ± 11.7	246 ± 10.7^C^
**Calories (Cal)**	395 ± 7.5 ^A,E^	378 ± 11.3 ^B,F^	355 ± 11.3 ^C^	628 ± 8.7 ^D,E,F^	845 ± 44.4 ^A,B,C,D^

Values are mean ± SEM for six rats per group. Means in a row with superscripts with a common letter differ, (small letter, *p* < 0.05 or capital letter, *p* < 0.01). CD, control diet; HSFD, high-saturated fat diet; HUFD, high-unsaturated fat diet; HDSD, high-digestible starch diet; HRSD, high-resistant starch diet. ANOVA and Bonferroni’s multiple comparison test.

**Table 3:** Effects of experimental diets on glycemic control parameters.

Variable	CD	HSFD	HUFD	HDSD	HRSD
**Glucose (mg/dL)**	108.2 ± 11.2	94 ± 5.9	83 ± 4.0 ^a^	98 ± 4.0	121 ± 3.0 ^a^
**Insulin (ng/mL)**	1.1 ± 0.2 ^a^	0.6 ± 0.2	0.2 ± 0.1 ^a,B^	1.3 ± 0.3 ^B^	0.9 ± 0.2
**HOMA Index**	7.5 ± 1.7 ^A^	3.8 ± 1.3	0.9 ± 0.3^ A,B,C^	7.8 ± 1.9 ^B^	6.6 ± 1.5 ^C^

Values are mean ± SEM, n = 6. Means in a row with superscripts with a common letter differ, (small letter, *p* < 0.05 or capital letter, *p* < 0.01). CD, control diet; HSFD, high-saturated fat diet; HUFD, high-unsaturated fat diet; HDSD, high-digestible starch diet; HRSD, high-resistant starch diet. ANOVA and Bonferroni’s multiple comparison test.

The lowest serum insulin levels were also observed in the HUFD which were significant in comparison with the CD and the HDSD. The highest insulin value was observed in the HDSD animals, however, it did not reach statistical significance in comparison with the CD but was significantly higher than the HUFD group (*p* < 0.05). As expected from the glycemia and insulin levels, insulin resistance estimated as HOMA-IR was the lowest in HUFD compared to CD, HDSD and HRSD groups (*p* < 0.05).

**Figure 2:**
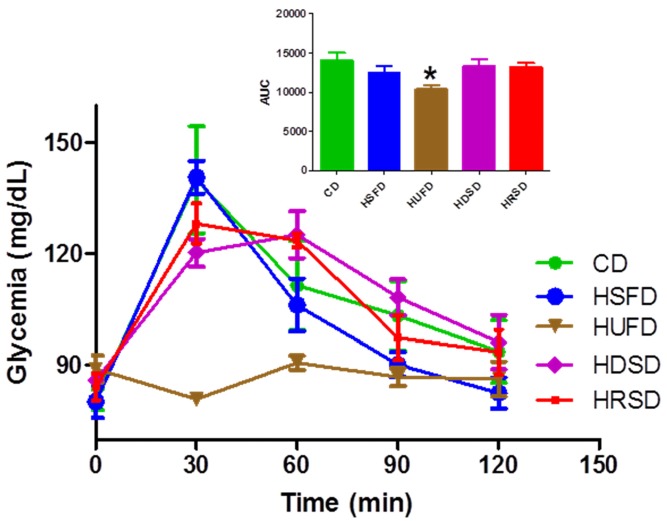
Glucose tolerance test results two days before sacrificing of the animals. Control diet (CD), high-saturated fat diet (HSFD), high-unsaturated fat diet (HUFD), high-digestible starch diet (HDSD), high-resistant starch diet (HRSD). AUC HUFD *vs.* AUC other groups, *p* < 0.001. ANOVA and Bonferroni’s multiple comparison test.

Results from the OGTT test performed at the end of the experimental period are shown in Figure 2. No significant differences were observed between the glucose AUCs from the HSFD, HDSD, and HRSD compared to the CD group. However, the HUFD curve showed a low 30-min glucose concentration having a lower AUC in comparison with the CD and the other groups (*p* < 0.001). These findings were confirmed by repeating the determinations and checking the correct functioning of the glucometer during the OGTT performance.

### 3.3. Lipidic Control

Table 4 shows the results of variables related to the lipidic control. In spite of the observed higher caloric intake, HRSD presented lower levels in serum triglycerides (TAG), cholesterol and HDL-Chol in comparison with the CD and HDSD (*p* < 0.05 and *p* < 0.01, respectively). The major TAG levels were observed after HDSD treatment being higher than the two HFDs (*p* < 0.05) and HRSD (*p* < 0.01), however, it was not different to CD. Other groups, apart from HRSD, did not show differences in serum cholesterol and HDL-Chol concentration with CD or between them.

**Table 4:** Effects of experimental diets on serum lipids.

Variable	CD	HSFD	HUFD	HDSD	HRSD
**TAG (mg/dL)**	118 ± 17.1 ^a^	67 ± 6.8 ^c^	65 ± 9.2	147 ± 17.9 ^B,c^	55 ±9.4 ^a,B^
**Chol (mg/dL)**	73 ± 4.1	73 ± 4.5	81 ± 3.3 ^A^	70 ± 4.9	50 ± 2.6^A^
**HDL-Chol (mg/dL)**	30 ± 1.2	34 ± 1.9 ^b^	35 ± 1.6 ^a^	31 ± 2.0	26 ± 0.8 ^a,b^

Values are mean ± SEM, n = 6. Means in a row with superscripts with a common letter differ, (small letter, *p* < 0.05 or capital letter, *p* < 0.01). CD, control diet; HSFD, high-saturated fat diet; HUFD, high-unsaturated fat diet; HDSD, high-digestible starch diet; HRSD, high-resistant starch diet. ANOVA and Bonferroni’s multiple comparison test.

### 3.4. Hepatic Determinations

No differences on total liver weight were observed between the groups after the experimental period (data not shown). Table 5 shows that liver total lipids in HFDs increased compared with other groups, however, only the HUFD group reached significance respect to CD, HDSD, and HRSD groups (*p* < 0.01). The liver total lipids in the HSFD group increased significantly only in comparison with the HRSD group (*p* < 0.01). There were not differences in HDSD and HRSD compared with the CD group.

**Table 5:** Effects ofexperimental diets on hepatic lipids.

Variable (mg/g)	CD	HSFD	HUFD	HDSD	HRSD
**Total Lipids **	36 ± 1.3 ^A^	50 ± 3.4 ^B^	58 ± 2.3 ^A,C,D^	39 ± 3.3 ^D^	29 ± 0.7 ^B,C^
**CHOL **	2.1 ± 0.08	3.0 ± 0.07 ^a^	2.2 ± 0.1	3.0 ± 0.2^ b^	1.9 ± 0.09 ^a,b^
**TAG**	2.9 ± 0.2 ^c,d^	3.6 ± 0.6 ^a,d,f^	2.8 ± 0.4 ^a,b^	3.7 ± 1.1 ^b,c,e^	2.9 ± 0.4 ^e,f^

Values are means ± SEM, n = 6. Means in a row with superscripts with a common letter differ, (small letter, *p* < 0.05 or capital letter, *p* < 0.01). CD, control diet; HSFD, high-saturated fat diet; HUFD, high-unsaturated fat diet; HDSD, high-digestible starch diet; HRSD, high-resistant starch diet. ANOVA and Bonferroni’s multiple comparison test.

Hepatic cholesterol levels increased after the HSFD and the HDSD treatments, however did not reach significant statistical difference from the CD group. On the other hand the lowest cholesterol value was observed in the HRSD group which was significantly lower in comparison with HSFD and HDSD groups.

Hepatic triglyceride levels were significantly higher in the HSFD and the HDSD groups in comparison with the CD group (*p* < 0.05); also HSFD was higher than HUFD (*p* < 0.05) and HDSD was higher than the HRSD group (*p* < 0.05). HRSD and HUFD triglyceride levels were not different from the values in CD group.

## 4. Discussion

Most experiments in nutrition research have analyzed the differential effects of high-fat diets on metabolic control, but few studies have focused on complex carbohydrates. Typically diets containing simple carbohydrates like sucrose are compared with diets containing digestible starch. In the present study, the effects of two different HCDs and two HFDs on energy balance, metabolic control and insulin resistance in a group of normal Wistar rats were analyzed in comparison with rats fed a normal standard diet. To achieve this goal a group of animals fed a high-resistant starch diet in comparison with a high-digestible-starch diet and the HFDs were included. In previous experiments our group showed the beneficial effects of the native banana starch (NBS) used in this study improving metabolic control, reducing blood lipids and body weight in rats as well as in obese subjects [19,20,21].

This study was performed in normal growing rats (7 weeks of age) in the middle of the adolescence to the adult period. Hence, it might be supposed that the reduction in body weight gain observed in the experimental diets compared to CD group, primarily reflect the reduction of growth rather than reduction of obesity since the animals were not obese. The body weight AUCs did not show significant differences, even though the rats fed the HCDs exhibited higher caloric consumption, HRSD group with more than 200% and the HDSD group with an energy intake of more than 50% compared with the other groups. At the end of the experimental period the reduction of body weight gain was more moderate in the HDSD group. However, comparison in food consumption with the CD group must be interpreted with care because the preparation of the diets. The CD was administered as pellets as purchased from Harlan laboratories, however, all the other diets were daily freshly prepared as pellets. Thus, differences in the consistency of the food could have introduced differences in food consumption when compared to CD group. Of note, HDSD final body weight was not statistically different from the CD group. These findings cannot be attributed to the high-calorie consumption since the HRSD had higher consumption than the CD group but lower body weight gain. This moderate differential effect on body weight in the HCDs, might be partially explained by an increased accumulation of body fat, however, this was not measured in this study.

As mentioned above, energy intake in this study was measured by the difference among the daily food weights and resulted in values between 1.1 and 4.2 g/day which might appear at first sight too high to reach, in special in the case of the HRSD group which observed the highest values. However, this can be partially explained by the reduced energy density of this diet in comparison with the HCDs. Moreover, it should be noted that, due to its resistance to digestion, the caloric availability of RS is even lower than that for digestible starches. Most digestible carbohydrates provide 4 Kcal of available energy/g (value used in Table 1) whereas RS provides 2–3 Kcal/g. Considering that NBS contains only 34% of resistant starch, the corrected caloric density of the HRSD could be near to 3.6 Kcal/g.

Besides, another mechanism to explain the difference in the energy intake consumption between the HFDs and the HCDs might be the higher satiating effect of the HFDs because of their slower digestion in comparison with the HCDs rendering rats more efficient with their ingested calories [24,25]. However, the anorexigenic effect of some low-carbohydrate diets mediated by the production of ketones was not expected in this study because the carbohydrates content in these diets are not enough to induce ketogenesis. On the other hand, the high consumption of NBS observed in the HRSD group here, could be in contrast with others who have informed that a high amylose diet reduce energy intake in obese rats fed *ad libitum* in comparison with a high amylopectin diet [26]. Others have also informed beneficial effects of resistant starch in humans increasing satiety [27,28]. This findings warrant further studies to determine NBS satiety and satiation since the current study was not designed for that purpose. Studies in humans have demonstrated that both of them HFDs or HCDs reduces body weight similarly. However, in some cases, the HFDs have been found to exhibit metabolic disadvantages that may offset the benefits of weight reduction [29,30].

When comparing HRSD *vs.* HDSD no significant differences were observed on the glycemic control parameters, however, the differential effects of the HFDs was clearly manifest. Findings from the OGTT performed two days before the end of the experimental period showed that the highest glucose tolerance was found in HUFD-fed animals. Moreover, further basal determinations performed two days after the OGTT confirmed that HUFD group exhibited the lowest fasting glycemia and insulin levels. Thus, HUFD group was the best diet to improve glucose tolerance and fasting glycemia.

The beneficial effects of HUFDs may be partially explained by the olive oil content in this diet. In addition to monounsaturated fatty acid (MUFA) and polyunsaturated fatty acid (PUFA), olive oil contains tocopherols, carotenes, and other phenolic compounds which exhibit antioxidant properties [31,32]. Potential mechanisms of action of olive oil are the augmentation of gastric emptying, reducing the glucose absorption and the increased insulin sensitivity by improving insulin-receptor union, cellular permeability and signaling [33]. Prieto *et al.* reported that olive oil supplementation improved glucose homeostasis and increased glucagon-like peptide-1 (GLP-1) in rats [34]. In humans, Garg *et al.* reported that a high-monounsaturated-fat diet provided lowered plasma glycemia in type 2 diabetics when compared to a HCD [35]. These olive oil favorable effects on glucose homeostasis involve the increased secretion of GLP-1 [36].

A few years ago, postprandial glycemia peaks were only attributed to the simple carbohydrates like mono- or di-saccharides as fructose or sucrose, however, in recent years it has been known that some complex carbohydrates may exhibit the same hyperglycemic peaks when they are digested rapidly [37]. The rapid catabolism of highly refined corn starch used in the formulation of HDSD might explain the high levels of fasting insulin and fasting glycemia found after treatment. On the other hand, the native banana starch (NBS) used in this study has demonstrated beneficial effects on glycemic and insulin response in animal models as well as in diabetic subjects [19,20,21]. The beneficial effects of NBS could be attributed to the short chain fatty acid production during the colonic fermentation, being these acids associated with the increased production of GLP-1, cholecystokinin, adiponectin, leptin and other incretins [38].

Like the observed effects of the different components of high-fat diets to modify glycemia control parameters, the differential effects of the HCDs were more important on serum and hepatic lipids. Although, we expected that HFDs did increased serum TAG or cholesterol levels no changes were observed compared to the CD group. However, the HDSD increased serum and hepatic TAGs concentration whereas the HRSD lowered both of them (*p* < 0.05). The HRSD group exhibits also a tendency to reduce serum cholesterol and HDL-cholesterol levels as well as a tendency to reduce hepatic cholesterol. Taken together these results suggest that the composition of the high-carbohydrate diet is as important as the composition of the high-fat diets to affect metabolic control and insulin resistance. Further longer studies using NBS and an unsaturated-fat diet are needed in other animal models before extrapolating these results to subjects at high risk in order to prevent or reduce chronic complications by improving metabolic control and insulin resistance. On the other hand, our results confirm the tight interaction between carbohydrate and lipid metabolism. Any problem arising in either one of these processes almost invariably results in serious changes in the other metabolic pathways.

Limitations of the present study include the lack of some measure of body adiposity which would have given the possibility to observe whether the differences in body weight were related to changes in body fat. Moreover, the differences between food consumption HCDs and HFDs effects might be skewed by a little difference in the protein content between these diets, in the case of HFDs there is a 18.6% protein but for HCDs a 21.6% was used. 

## 5. Conclusions

In conclusion, after an 8-week administration, HFD and the HCD produced similar effects in reducing body weight gain, in spite of the different caloric consumption. The most beneficial effects on glycemic control were observed after the consumption of HUFD, whereas the HRSD exhibited the most anti-atherogenic effects in reducing serum and hepatic lipids. These findings are a manifestation of the complex interrelation between carbohydrates and lipid metabolism, since it is impossible to alter one of them without changing the other. In addition, not only fat composition is important because its deleterious effect on glycemic and lipidic control but the type of carbohydrate is determinant on the metabolic control.
